# Real-World LEAP Implementation

**DOI:** 10.1007/s11882-022-01032-3

**Published:** 2022-04-08

**Authors:** Jennifer J. Koplin, Victoria X. Soriano, Rachel L. Peters

**Affiliations:** 1 Murdoch Children’s Research Institute, Royal Children’s Hospital, 50 Flemington Rd, Parkville, VIC 3052 Australia; 2 Department of Paediatrics, University of Melbourne, Parkville, VIC Australia

**Keywords:** Food allergy, Prevention, Early introduction, Peanut allergy, Egg allergy, Complementary foods

## Abstract

***Purpose of Review*:**

In 2015, findings from the Learning Early About Peanut allergy (LEAP) trial provided the first convincing evidence that peanut allergy may be preventable through early peanut introduction into the infant diet. Here we discuss implementation of the LEAP study findings around the world and emerging evidence of the impacts on infant feeding and food allergy.

***Recent Findings*:**

The LEAP findings led to rapid changes in allergy prevention guidelines internationally to recommend early peanut introduction. There is now emerging evidence that this has been followed by a substantial increase in early peanut introduction to infants. Studies investigating the impact of these changes in infant feeding practices on the prevalence of peanut allergy are underway.

***Summary*:**

The LEAP trial represented a significant step forwards in food allergy prevention and new research over the past 5 years has provided insights into how best to implement this intervention in the real world.

## Introduction

In 2015, findings from the Learning Early About Peanut allergy (LEAP) randomized controlled trial provided the first convincing evidence that peanut allergy may be preventable [1]. The LEAP study showed a substantial reduction in peanut allergy among children with eczema and/or egg allergy by introducing peanut in the first year of life (between 4 and 11 months of age), compared with complete peanut avoidance until age 5 years. Despite the paradigm shift that this represented, multiple unanswered questions remained about how best to implement this intervention in the real world. The past 5 years has provided further insights into many of these questions. Here we discuss approaches to implementation of the LEAP study findings around the world and emerging evidence of the impact of these approaches on infant feeding and food allergy. We also discuss potential next steps in food allergy prevention in the post-LEAP era.

## Implementation of Early Allergen Introduction—International Guidelines

The publication of the LEAP findings led to rapid changes in infant feeding and allergy prevention guidelines around the world to recommend early peanut introduction, although specific advice differed between countries. In particular, advice differed with regard to recommended age for first peanut introduction, whether advice was specifically targeted to high-risk groups or to the general population, and whether “high-risk” infants should undergo screening prior to first peanut ingestion. Some guidelines focused only on early peanut introduction, while others also recommended early introduction of egg, which was shown in meta-analyses of randomized controlled trials to reduce the risk of egg allergy [2]. Although there is currently insufficient evidence from randomized controlled trials to guide recommendations around introduction of other allergenic foods such as tree nuts, several guidelines recommend introduction of a wide variety of allergenic foods in the first year of life based on the hypothesis of a similar mechanism of protection irrespective of the allergen in question.

In response to the LEAP findings, the Centre for Food and Allergy Research, the Australasian Society of Clinical Immunology and Allergy, and the Australian National Allergy Strategy hosted an Australian Infant Feeding Summit in 2015 to appraise the new evidence and developed revised recommendations for infant feeding [3]. Three recommendations resulted from the national consensus between experts, stakeholders, and researchers: (1) introduce solid foods around 6 months of age, but not before 4 months; (2) introduce allergenic foods including peanut butter and cooked egg in the first year of life; and (3) hydrolyzed infant formula is not recommended for the prevention of allergic disease. The consensus aimed to provide consistency between different Australian guidelines to ensure clear consumer advice while balancing the need for food allergy prevention with other nutritional priorities, including the known benefits of breastfeeding. These recommendations were incorporated into the Australasian Society of Clinical Immunology and Allergy’s infant feeding guidelines in 2016 [4].

In the USA, guidelines released shortly after publication of the LEAP trial initially provided three separate guidelines on peanut introduction depending on their potential risk level for developing peanut allergy. It was also recommended that infants at high risk should be tested or screened for peanut allergy prior to first peanut introduction [5]. This advice has now been superseded by consensus guidelines released by the American Academy of Allergy, Asthma, and Immunology; American College of Allergy, Asthma, and Immunology; and the Canadian Society for Allergy and Clinical Immunology in 2021 [6]. The new guidelines are consistent with the Australian consensus guidelines described above, and state that both peanut and egg should be introduced around 6 months of life, but not before 4 months; other allergens should be introduced around this time as well; and screening before introduction is no longer recommended.

The Asia Pacific Association of Pediatric Allergy, Respirology & Immunology (APAPARI) consensus statement published in 2017 differentiates between high and low peanut allergy prevalence countries, with early peanut introduction recommended only for high prevalence countries and high-risk infants [7]. Some Asian countries such as Singapore have a low prevalence of peanut allergy despite delayed introduction of peanut into the infant diet, potentially supporting different recommendations and implementation of the findings of the LEAP trial in different countries [8].

In 2021, the European Academy of Allergy and Clinical Immunology (EAACI) also published updated guidelines on food allergy prevention [9•]. These guidelines recommend introducing egg and peanut into the infant diet as part of complementary feeding, with peanut introduction recommended specifically for populations with a high prevalence of peanut allergy. The guidelines also suggest that the most effective age to introduce egg and peanut is from 4 to 6 months of life. The EAACI committee noted that evidence for a benefit of early peanut introduction is currently limited to the UK where the LEAP trial was conducted, a country with a high prevalence of peanut allergy. As a result, the EAACI guidelines made no recommendations on peanut introduction for countries with a low peanut allergy prevalence, due to concern that findings may not be generalizable to countries with a low prevalence of peanut allergy.

## Measuring Population Uptake of Early Allergen Introduction

Allergy prevention guidelines have undergone three major changes since the 1990s, from initially recommending dietary allergen avoidance in infancy to prevent food allergy, to removal of this advice around 2008, and subsequently the strong recommendation to introduce allergens in the first year of life to prevent allergy which started to be incorporated in guidelines from around 2016. Given this major shift in approach, it would not be surprising if parents and healthcare providers expressed some skepticism and reluctance to follow the latest advice. Early surveys in the USA noted low willingness to implement early allergen introduction among both parents and healthcare providers [10], adding to these concerns. We also showed in a large population-based Australian cohort that removal of advice to delay allergen introduction in 2008 was followed by only a small shift towards earlier introduction of peanut, with the majority of parents continuing to avoid giving peanut products to infants throughout the first year of life [11].

Despite these early concerns, we recently demonstrated high uptake of early peanut introduction among Australia infants after publication of the LEAP trial and the 2016 guideline changes. We conducted two large population-based studies in Melbourne, Australia, 10 years apart, including a total of over 7,000 participants, using the same sampling frame and methods to assess the impact of infant feeding guidelines on egg and peanut introduction as well as food allergy outcomes. We showed a striking shift towards earlier peanut introduction, with a threefold increase in peanut introduction by age 1 year in 2018–2019 compared with 2007–2011 [12••]. Peanut introduction in the first year of life increased from 28 to 88%. Infants at high risk of food allergy, namely those with early-onset eczema, had similarly high rates of early peanut introduction, providing reassurance that these guidelines are being taken up by the group who are likely to benefit the most from early peanut introduction. These findings were supported by a national survey of 1940 parents, which showed similarly high rates of peanut introduction in infants by age 12 months (86%) [13].

While the finding that families in Australia have responded positively to updated infant feeding guidelines are promising, it remains important to measure uptake of early allergen introduction in other countries with a high prevalence of food allergy. Additionally, investigation of whether the recent changes to US guidelines affect parent and health care provider willingness to implement early allergen introduction is warranted. Finally, the critical unanswered question is whether the changes in infant feeding practices have resulted in a decrease in prevalence of food allergy.

## Impact on Allergy Prevalence

The LEAP trial, as a single randomized controlled trial conducted in a select group of high-risk infants, was unable to answer the question of what proportion of peanut allergy in the general population is preventable through early peanut introduction. Only infants with eczema or egg allergy were eligible; thus, no information is available for whether earlier introduction of peanut also reduces the risk of peanut allergy in infants without these risk factors. Infants also underwent skin prick test screening at study entry and only those with a wheal < 4 mm to peanut were included, further reducing the generalizability of the findings. In addition, the trial involved an intensive, long-term intervention (daily peanut consumption at relatively high doses starting in infancy and continuing to 5 years of age). Compliance was very high, likely due to regular contact and assistance from study staff and the inclusion of a very motivated group of parents. It is not clear whether this dose and frequency will be achieved by parents in the community, and whether smaller, less frequent doses as might be given in the real-world setting are also effective. In 2016, we attempted to answer some of these questions using data from the HealthNuts study, a population-based cohort of 5300 infants in Australia, to understand the implications and generalizability of the LEAP trial’s findings regarding the introduction of peanut at the population level [14]. Our modelling suggested that if the LEAP intervention findings were applied to the general population, between 44 and 64% of peanut allergy cases might be preventable. The lower estimate assumes that early peanut introduction is only effective in infants eligible for inclusion in the LEAP trial, i.e. those with early onset eczema and/or egg allergy. The higher estimate assumes the timely peanut introduction was equally effective in preventing peanut allergy in infants without these risk factors as well as in the approximately 20% of infants with a SPT wheal size more than 4 mm who do not have a pre-existing peanut allergy.

More recently, we investigated the impact of earlier peanut introduction on the prevalence of peanut allergy using data from two large population-based studies in Melbourne, Australia, conducted 10 years apart using the same sampling frame and methods and including a total of over 7,000 participants. Peanut introduction in the first year of life increased from 28 to 88% over this time period [12••]. Despite this, we found only a relatively small reduction in peanut allergy prevalence, from 3.1 to 2.6%, highlighting that more needs to be done to prevent peanut allergy in the general population [15].

## Impact of Early Allergen Introduction on Other Outcomes

Despite the benefits of early allergen introduction on food allergy prevention, changes to infant feeding guidelines to recommend early allergen introduction may have unintended consequences on other health outcomes, which clinicians and public health advisors should be mindful of. We recently observed an increase in hospital presentations for nut inhalation over a 10-year period, with the increase most marked since 2015, after the publication of LEAP [16•]. The increase was most notable in infants and young children (< 3 years). Although presentations to hospital with nut inhalation remain exceedingly rare, these findings emphasize the importance of promoting safe introduction of hard foods in young children when recommending early peanut introduction.

Because of the known benefits of breastfeeding to both infants and mothers, weaning advice needs to balance the need to promote breastfeeding with allergy prevention. Reassuringly, there is some evidence from both randomized controlled trials and population-based studies that earlier introduction of allergenic solids has not had a detrimental impact on breastfeeding rates [17, 18].

Another outcome that may be affected by age at introduction of allergenic foods is growth in childhood. There is low to moderate quality evidence that introducing solids prior to age 4 months can increase the risk of overweight and obesity in childhood [19]. Reassuringly, we recently showed that in Australia, the introduction of guidelines recommending early allergen introduction did not lead to an increase in introduction of solid foods before 4 months of age [18]. There was also concern that introduction of peanut products, which are often high in calories, to infants could increase the risk of children becoming overweight; however, the LEAP trial found no evidence that this was the case [20].

Guidelines need to consider and carefully balance the wide range of health outcomes that may be associated with timing of introduction of allergenic foods. It is important that research continues to measure the longer-term impacts of early allergen introduction on both food allergy and other health outcomes to ensure that early allergen introduction for food allergy prevention is not associated with any unintended detrimental impacts on other health outcomes.

## Potential Impact of the COVID Pandemic

The COVID pandemic has had major impacts on healthcare internationally. In Australia, the pandemic led to removal of face-to-face appointments with Maternal and Child Health nurses, a key source of infant feeding information [12••]. This may have reduced the previously high uptake of early peanut introduction. In addition, increased microbial exposure has been associated with a reduced risk of food allergy [21]. Increased hand sanitization, closures of playgrounds, childcares, and social isolation due to the covid-19 pandemic and associated restrictions are likely to have had a striking impact on microbial exposure of infants at a critical period of immune system development. We hypothesize that these factors might lead to a subsequent increase in food allergy prevalence. It will be important to continue to monitor both uptake of infant feeding advice around early introduction of allergenic foods and food allergy prevalence post-pandemic.

## Other Prevention Strategies

Collectively, current evidence indicates that more will need to be done to prevent the development of food allergy, even if optimal introduction of allergenic foods to infants in the first year of life can be implemented for all infants. In LEAP, around 9% of infants who were screened were excluded from the trial because their SPT wheal was ≥ 4 mm, suggesting the infant was already likely to have peanut allergy. Likewise, in RCTs on early introduction of egg, some infants had allergic reactions on their first known exposure to egg even when this occurred early in the first year of life [1, 22]. Therefore, some infants may develop food allergy before they are developmentally ready to consume solid foods. Multiple other strategies for food allergy prevention are currently under investigation, with several being tested in large-scale randomized controlled trials.

### Eczema

Eczema is perhaps the strongest known risk factor for food allergy, with at least a six-fold increase in food allergy among infants with eczema compared to those without [23]. The risk of food allergy also increases with earlier onset of eczema and with increasing severity [23]. Food allergy is relatively uncommon in children without any eczema in the first year of life. The mechanisms driving this association between eczema and food allergy have not been conclusively established, although it has been hypothesized that food sensitization leading to food allergy could occur through a damaged skin barrier in eczematous skin, and this hypothesis is supported by animal studies and some observational data [24]. Other potential explanations include shared genetic or environmental risk factors for both conditions. Several studies have attempted to prevent eczema in early life, with some of these also aiming to prevent food allergy by reducing eczema. Although early small studies showed promising reductions in eczema with regular use of emollients starting in early infancy [25], more recent larger trials of different skin interventions using petroleum-based emollients (PreventADALL and BEEP) have been unsuccessful in preventing eczema [26, 27]. Research suggests that skin with atopic dermatitis tends to have decreased levels of ceramides. Therefore, moisturizers containing ceramides may provide a more effective protection against eczema. We await the results of a further ongoing study of early ceramide-containing emollients for prevention of eczema and food which showed promising results in a pilot study [28].

### Maternal Consumption of Allergenic Foods During Pregnancy and Breastfeeding

As described above, timely introduction of allergenic foods into the infant diet alone is insufficient to prevent food allergy in all infants. Some infants may have allergic reactions on first introduction of egg or peanut as early as 4 months of age and are therefore unable to benefit from “early” introduction of these foods into their diet [1, 22]. Consumption of these foods by the mother during pregnancy and breastfeeding at high doses may offer an alternative route of early exposure. To date, the role of maternal consumption of allergenic foods in the risk of food allergy has mostly been investigated in observational studies, with inconsistent findings. An ongoing large-scale randomized controlled trial (PrEggNut) now aims to conclusively determine whether a high egg and peanut maternal diet reduces the risk of challenge-confirmed egg and peanut allergy in offspring compared to a standard maternal diet [29].

### Vitamin D

Ecological and epidemiological evidence suggests a link between low vitamin D levels and an increased risk of food allergy. Data from observational studies has however been inconsistent and large-scale randomized controlled trials of infant vitamin D supplementation for food allergy prevention are lacking. The ongoing VITALITY randomized controlled trial aims to fill this evidence gap, with results expected in 2022 [30].

### Early Introduction of Other Allergenic Foods

There is currently insufficient evidence to specifically guide advice around introduction of other allergenic foods such as tree nuts into the infant diet, although several studies are currently underway investigating this issue. It certainly seems plausible that similar mechanisms of protection may be present for tree nut as for peanut allergy. However, it is also possible that different “windows of opportunity” exist for different foods. One notable example is cow’s milk, with a recent RCT showing a reduction in cow’s milk allergy at age 6 months with daily cow’s milk formula ingestion from 1–2 months of age compared with cow’s milk avoidance during this early life period [31]. It is however notable that rates of cow’s milk formula supplementation in the first weeks of life, prior to the intervention, were high in both groups, and it is possible that higher rates of cow’s milk allergy in the avoidance group are being driven by early exposure followed by a period of avoidance of cow’s milk, rather than by delayed introduction per se [32]. The recent EAACI guidelines recommend avoiding the use of regular cow’s milk formula as supplementary feed for breastfed infants in the first week of life, based on low certainty evidence from a single trial that this may reduce the risk of cow’s milk allergy [9•]. Further high-quality trials are needed in this space.

## Conclusions

The LEAP trial represented a significant step forwards in food allergy prevention and new research over the past 5 years has provided insights into how best to implement this intervention in the real world. Allergy prevention guidelines around the world now recommend early peanut introduction into the infant diet to reduce the risk of peanut allergy developing. There is emerging evidence from some countries that the introduction of these guidelines was followed by a substantial increase in early peanut introduction to infants, and studies investigating the impact of these changes in infant feeding practices on the prevalence of peanut allergy are underway. Ongoing research is also required to ensure there are no unintended consequences of recommending early allergen introduction on other health outcomes. It is also clear that additional prevention strategies will be needed to prevent food allergy in infants who fail to benefit from early allergen introduction, and we eagerly await the results of several ongoing large-scale trials testing novel food allergy prevention strategies.
